# Inverse Association between Prediagnostic IgE Levels and the Risk of Brain Tumors: A Systematic Review and Meta-Analysis

**DOI:** 10.1155/2015/294213

**Published:** 2015-09-13

**Authors:** Chong Ma, Lei Cao, Jianping Zhao, Xing Ming, Ming Shang, Hailiang Zong, Hai Du, Kai Li, Xiaoguang He, Hongsheng Xu

**Affiliations:** Department of Neurosurgery, Central Hospital of Xuzhou, Affiliated Hospital of Southeast University, Xuzhou, Jiangsu 221009, China

## Abstract

An inverse association between allergic conditions and glioma risk has been suggested in many epidemiological studies. However, the evidence is inadequate to draw robust conclusions for the association between prediagnostic IgE levels and brain tumors risk. The aim of this study was to provide more precise estimates for this association by meta-analysis of all published studies. Overall, 8 individual studies with 2,461 cases and 3,934 controls were included in our study. A decreased risk of brain tumors (RR = 0.73, 95% CI 0.61–0.86, *P* < 0.001) was observed in relation to elevated level of total IgE. The negative association was significant between elevated total IgE level and the risk of glioma (RR = 0.74, 95% CI 0.62–0.88, *P* = 0.001). However, no significant relationship was demonstrated between testing positive for respiratory allergen-specific IgE and brain tumors risk. In addition, the role of prediagnostic IgE levels in brain tumors risk did not alter in men and women. The present study suggests that increased level of total prediagnostic IgE but not respiratory allergen-specific IgE plays a protective role in brain tumors risk, glioma in particular. More studies are warranted for further elucidation of the meningioma risk related to prediagnostic IgE levels.

## 1. Introduction

Glioma and meningioma are two common primary brain tumors in adults [1]. Glioma is the most common type representing more than 80% of adult brain tumors [2]. Meningiomas are primarily benign tumors derived from meningothelial cells of the arachnoid membrane [3]. Ionizing radiation and genetic predisposition are well established risk factors for brain tumors [4–6]. However, little is known about the etiology of brain tumors.

The link between allergy and brain tumorigenesis is attracting much attention but remains largely unknown. Allergy is composed of eczema, hay fever, allergic asthma, and other heterogeneous diseases with complicated mechanisms. Some common allergies are characterized by immediate hypersensitivity reactions and mediated by immunoglobulin E (IgE) generated by B cells as well as T helper cells [7, 8]. IgE is a prediagnostic biomarker of allergy [9, 10]. Increased serum IgE is a powerful indication for allergic diseases. Both total serum IgE and allergen-specific IgE participate in the allergic response. Specific serum IgE is indicative of allergic sensitization to specific allergens of respiratory tract, food, or other origins. It is hypothesized that a highly active immune system leads to an enhanced tumor immune surveillance through recognizing and killing tumor cells. Whether prediagnostic IgE levels could modify the risk of brain tumors is currently unclear due to inconsistent and inconclusive findings in previous epidemiological studies. We aim to present more precise estimates for roles of prediagnostic total IgE and respiratory allergen-specific IgE levels in brain tumorigenesis by performing a meta-analysis of all published studies.

## 2. Materials and Methods

### 2.1. Search Strategy

A comprehensive literature search was performed in PubMed and Embase databases for eligible studies on the relationship between prediagnostic IgE levels and brain tumors risk. The last search was on June 26, 2014. The following terms were used: immunoglobulin E, IgE, total IgE level, respiratory allergen-specific IgE level, allergic marker, or allergy and brain tumors, brain cancer, glioma, glioblastoma, or meningioma. The references of retrieved studies were also screened for other relevant articles. No language restriction was imposed.

### 2.2. Inclusion Criteria

Studies included into our study have to meet the following inclusion criteria: (1) studies on the relationship between prediagnostic IgE levels and brain tumors risk; (2) studies in case-control or cohort design; and (3) studies presenting odds ratio (ORs), relative risks (RRs), or hazard ratios (HRs) with corresponding 95% confidence intervals (95% CIs) for association estimates. Case-only design, case reports, systematic reviews, meta-analysis, animal studies, or studies with duplicated data were all excluded.

### 2.3. Data Extraction

Two investigators independently extracted data from each eligible study. The following information was extracted: name of first author, publication year, country of origin, characteristics of subjects, study design, type of brain tumors, number of cases and controls, matching criteria, study period, adjusted factors, RRs or HRs or ORs with 95% CIs for assessment of prediagnostic IgE levels, and type of brain tumors. Disagreements on all terms were resolved by discussion.

### 2.4. Statistical Analysis

The association between prediagnostic IgE levels and brain tumors risk was estimated by calculating the pooled RRs with 95% CIs. Cochran's *Q*-statistic test and *I* ^2^ test were performed to evaluate the between-study heterogeneity, and *P* < 0.05 plus *I* ^2^ > 50% implicated significant between-study heterogeneity among all included studies [11, 12]. The random-effects model by the DerSimonian and Laird method was adopted when the between-study heterogeneity was significant [13]; otherwise, the fixed-effects model by the method of Mantel-Haenszel was used [14]. Stratified analyses by gender and type of brain tumors were also performed. Sensitivity analysis by sequentially omitting single studies one at a time was also carried out to assess the association. Publication bias risk was estimated by Begg's funnel plots and Egger's test [15, 16]. All analyses were performed by use of STATA 12.0 software (StataCorp, College Station, TX, USA). *P* < 0.05 suggested statistical significance.

## 3. Results

### 3.1. Characteristics of Studies Included into the Present Meta-Analysis

After a comprehensive literature search, we identified 8 independent studies on the association between prediagnostic IgE levels and brain tumors risk with a total of 2,461 cases and 3,934 controls [17–23].  Table 1 summarized the characteristics of all included studies. The studies were published between 2004 and 2013, which were performed primarily in USA and some European countries including Norway. Among the 8 studies, 6 were about the risk of glioma related to prediagnostic IgE levels, while the other 2 were regarding the meningioma risk.

### 3.2. Association between Total IgE Level and Brain Tumors Risk

**Figure 1:**
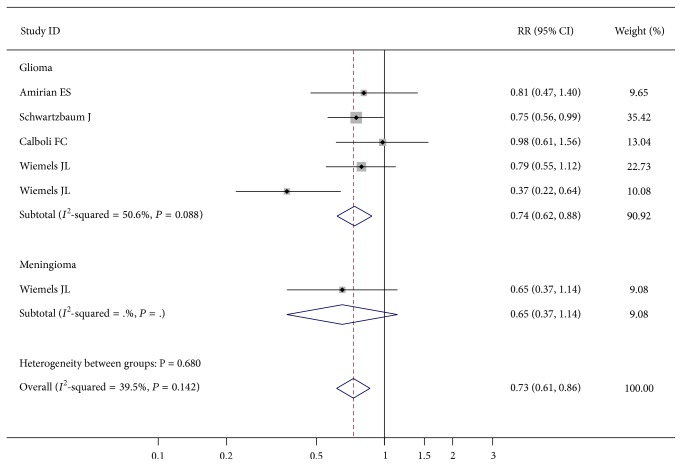
Forest plot for total IgE level and brain tumors risk.

The pooled RRs showed that elevated level of total IgE was associated with a decreased risk of brain tumors (RR = 0.73; 95% CI 0.61–0.86; *P* < 0.001) (Table 2, Figure 1). Besides, elevated total IgE level was negatively related to the risk of glioma (RR = 0.74; 95% CI 0.62–0.88; *P* = 0.001) (Table 2). Sensitivity analysis did not materially alter the combined results (data not shown).

### 3.3. Association between Respiratory Allergen-Specific IgE Level and Brain Tumors Risk

**Figure 2:**
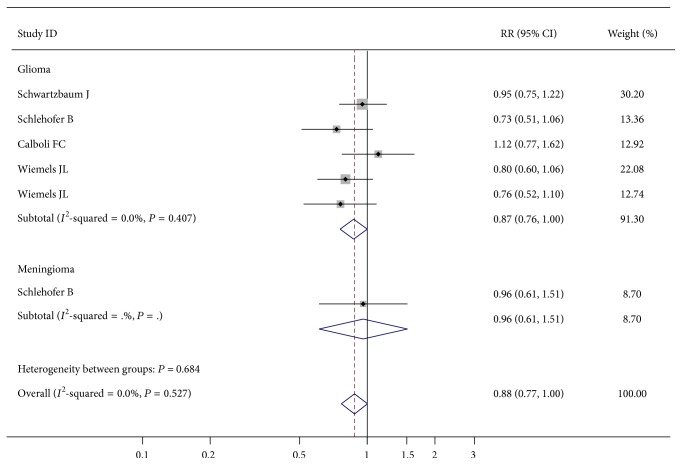
Forest plot for respiratory allergen-specific IgE level and brain tumors risk.

No significant association was observed between testing positive for respiratory allergen-specific IgE and brain tumors risk (Table 2, Figure 2). Sensitivity analysis confirmed the pooled results (data not shown).

### 3.4. Stratified Analysis by Gender

As shown in Table 2, no significant relationship of prediagnostic IgE levels (total IgE level and respiratory allergen-specific IgE level) with the risk of overall brain tumors was demonstrated among either men or women. Additionally, the role of prediagnostic IgE levels in glioma development did not change by gender, as suggested by stratified analysis by type of brain tumors in men and women, respectively (Table 2).

### 3.5. Heterogeneity Analysis and Publication Bias Risk

Results of Cochran's *Q*-statistic test and *I* ^2^ test were presented in Table 2 detailedly. There was no between-study heterogeneity no matter in overall analysis or stratified analyses by type of brain tumors and gender (Table 2). Begg's funnel plots and Egger's test implicated no potential publication bias in our study (data not shown).

## 4. Discussion

Common allergies consist of eczema, hay fever, and allergic asthma mediated by hypersensitivity reactions and high serum IgE concentrations. However, not all allergic individuals are characteristic of high IgE levels, and increased level of serum IgE cannot reflect all allergic diseases. The modifying effects of prediagnostic IgE levels on diseases initiation and progression alter among different diseases. Epidemiological studies have suggested inverse association between allergic diseases and malignant tumors [10, 24]. Self-reported allergies were shown to be associated with reduced risk of pancreatic cancer [25]. Allergy seems to be strongly and inversely related to childhood non-Hodgkin's lymphomas, as suggested by a recent pooled analysis [26]. Taken together, hypersensitivity was associated with reduced risk of malignancies, implicating an immune surveillance theory in carcinogenesis. Quite the reverse, there was no epidemiological support for the reverse association between allergic diseases and the risk of breast, prostate, and colorectal cancer [27]. Interestingly, a positive relationship between atopy and prostate cancer, but not breast and colorectal cancers, was demonstrated in that study [27]. Thus, despite extensive research, findings for allergy conditions and tumorigenesis warrant further elucidation.

IgE is a critical atopic marker linking allergy and cancer. Jensen-Jarolim et al. elaborated an evolving new field called AllergoOncology, which gave new insights into the role of IgE-mediated allergy in malignancies [28]. Due to its capacity of destroying tumor cells, IgE antibodies specifically targeting overexpressed tumor antigens have been identified as useful immunological agents. Besides, IgE nonspecifically binding to tumor cells has also been demonstrated to be a powerful adjuvant establishing tumor-specific immune memory [29, 30]. Moreover, IgE antibodies not only play critical roles in natural tumor surveillance, but also participate in active and adaptive immune responses involved in antitumor immunotherapy [28]. Additionally, macrophages, mast cells, and other IgE-receptor-expressed immune cells can become potent effectors in antitumor immunity; that is antitumor immunity by the bridge IgE. A number of epidemiological studies have been performed to estimate the association between prediagnostic IgE levels and brain tumors risk [17–23]. Nevertheless, the findings were inconsistent and inconclusive. Calboli et al. reported that total IgE levels were inversely associated with glioma risk [18]. However, no such association was observed for either respiratory allergen-specific or food allergen-specific IgE levels [18]. On the contrary, individuals with high levels of respiratory allergen-specific IgE were at decreased risk of glioma, but not meningioma [19]. As suggested by the study by Wiemels et al., increased serum total IgE concentrations were negatively related to the development of meningioma, indicating a protective role of atopic marker IgE in meningioma risk [23]. Taken together, the modifying effect of serum IgE level on brain tumors risk appears different with diverse types of brain cancer and the source of determined IgE. Up till now, no meta-analysis has been conducted to precisely estimate roles of prediagnostic IgE levels (total IgE level and/or allergen-specific IgE level) in brain tumorigenesis. A recent meta-analysis supported the evidence that allergic conditions were negatively related to the risk of glioma, suggesting a protective role of allergy in glioma development [31]. Nonetheless, the authors failed to assess the influence of specific allergies such as hay fever, eczema as well as allergic asthma, and allergic biomarker IgE in brain cancer risk. The association between different source of serum IgE and brain tumors risk, meningioma in particular, remains obscure and warrants further investigation. Our study firstly showed that increased level of total prediagnostic IgE but not respiratory allergen-specific IgE played a protective role in the risk of brain tumors, particularly glioma. It must be mentioned that the relationship of meningioma risk with prediagnostic IgE levels needs to be elucidated by more relevant epidemiological studies.

SNPs are supported to be important risk factors in brain tumorigenesis [5, 6, 32]. They can confer modifying effects on brain tumors risk independently or in combination with other factors, for instance, smoking and ionizing radiation. Interestingly, allergy-related SNPs can influence the development of brain tumors by interacting with immunological factors like prediagnostic IgE levels, which implicates critical roles of immune susceptibility factors in the etiology of brain cancers [32]. Gene polymorphisms of IL-4, IL-4R, and IL-13 represent promising immune factors in regulating IgE levels and tumorigenesis [32–34]. Unfortunately, we failed to investigate roles of such allergy-related SNPs in brain cancer risk in combination with prediagnostic IgE levels, in that very few studies have elucidated this issue up to date. The interaction between SNPs and serum IgE levels warrants further investigation to provide more support for the link between allergies and risk of brain tumors.

Findings in our study should be interpreted cautiously because of some limitations. Firstly, the strength of our study especially in relation to the meningioma risk was insufficient due to limited eligible studies published to date. Besides, only studies clearly presenting information about the detection of prediagnostic IgE levels were included into our study. More relevant studies with enough statistical power are encouraged in the future. Secondly, IgE levels were significantly associated with gender, age, smoking status, and ethnicity in glioma risk [21]. Apart from gender and type of brain tumors, we did not perform other stratified analyses by smoking, age, and so on, for lack of available published data. More studies with high quality are warranted for more precise estimates. Thirdly, inverse association was identified between elevated respiratory allergen-specific IgE level and high-grade glioma risk rather than low-grade glioma [19]. The effect of prediagnostic IgE levels on different subtypes of glioma was not estimated due to insufficient included publications. Lastly, the pooled analysis was based on unadjusted estimates, which might introduce bias. Some confounding factors including age, sex, IgE detection methods, smoking status, and education level of subjects should be considered in future studies.

## 5. Conclusions

A significant inverse association between total IgE levels and brain tumors risk is suggested in the present meta-analysis. The measurement of allergic biomarker IgE is valuable in targeting brain tumors, particularly glioma. In addition, the association between prediagnostic IgE levels and meningioma risk warrants further investigation. The study implicates that IgE monoclonal antibodies directing specifically against tumor-associated antigens can be a promising way of passive immunotherapy in brain cancer treatment.

## Figures and Tables

**Table 1:** Characteristics of all studies.

Study	Year	Brain tumors	Origins	Number of cases	Number of controls	Baseline time	Matching factors
Amirian et al. [17]	2013	Glioma	USA	362	462	2001–2006	Age, sex, and frequency
Schwartzbaum et al. [20]	2012	Glioma	Norway	594	1177	1974–2007	Date of blood collection, 2-year age interval at blood collection, and sex
Schlehofer et al. [19]	2011	Glioma	Europe	275	528	2002–2005	Study centre, gender, data of birth, age, date of blood collection, time of blood collection, and length of followup
Schlehofer et al. [19]	2011	Meningioma	Europe	175	343	2002–2005	Study centre, gender, data of birth, age, date of blood collection, time of blood collection, and length of followup
Calboli et al. [18]	2011	Glioma	USA	169	520	1976–2009	Age, age at blood draw, age at diagnosis, and ethnicity
Wiemels et al. [23]	2011	Meningioma	USA	265	145	2006–2009	Age, frequency, and state of residence
Wiemels et al. [22]	2009	Glioma	USA	393	470	2001–2004	Age, sex, ethnicity, and frequency
Wiemels et al. [21]	2004	Glioma	USA	228	289	1997–2000	Age, sex, ethnicity, and frequency

**Table 2:** Summary of meta-analysis results.

Comparisons	Number of studies	^ a^RR [95% CI]	^b^ *P* value	Tests for heterogeneity	
				*I* ^2^ (%)	^c^ *P*
*Total IgE level *					
Brain tumors	6	0.73 [0.61–0.86]	<0.001	39.5	0.142
Men	2	0.83 [0.63–1.10]	0.202	0.0	0.602
Women	2	0.69 [0.43–1.11]	0.125	0.0	0.450
Glioma	5	0.74 [0.62–0.88]	0.001	50.6	0.088
Men	2	0.83 [0.63–1.10]	0.202	0.0	0.602
Women	2	0.69 [0.43–1.11]	0.125	0.0	0.450
*Respiratory allergen-specific IgE level *					
Brain tumors	6	0.88 [0.77–1.00]	0.055	0.0	0.527
Men	4	0.96 [0.78–1.19]	0.744	0.0	0.770
Women	4	0.87 [0.67–1.15]	0.331	51.6	0.103
Glioma	5	0.87 [0.76–1.00]	0.051	0.0	0.407
Men	3	0.99 [0.80–1.23]	0.923	0.0	0.878
Women	3	0.81 [0.59–1.10]	0.172	60.3	0.081

^a^RR: relative risk; 95% CI: 95% confidence interval; ^b^ *P*: *P* values for pooled analysis; ^c^ *P*: *P* values for heterogeneity analysis.
